# Cognitive Impairment in Chronic Kidney Disease: Vascular Milieu and the Potential Therapeutic Role of Exercise

**DOI:** 10.1155/2017/2726369

**Published:** 2017-04-19

**Authors:** Ulf G. Bronas, Houry Puzantian, Mary Hannan

**Affiliations:** College of Nursing, Department of Biobehavioral Health Science, University of Illinois at Chicago, Chicago, IL, USA

## Abstract

Chronic kidney disease (CKD) is considered a model of accelerated aging. More specifically, CKD leads to reduced physical functioning and increased frailty, increased vascular dysfunction, vascular calcification and arterial stiffness, high levels of systemic inflammation, and oxidative stress, as well as increased cognitive impairment. Increasing evidence suggests that the cognitive impairment associated with CKD may be related to cerebral small vessel disease and overall impairment in white matter integrity. The triad of poor physical function, vascular dysfunction, and cognitive impairment places patients living with CKD at an increased risk for loss of independence, poor health-related quality of life, morbidity, and mortality. The purpose of this review is to discuss the available evidence of cerebrovascular-renal axis and its interconnection with early and accelerated cognitive impairment in patients with CKD and the plausible role of exercise as a therapeutic modality. Understanding the cerebrovascular-renal axis pathophysiological link and its interconnection with physical function is important for clinicians in order to minimize the risk of loss of independence and improve quality of life in patients with CKD.

## 1. Introduction

Chronic kidney disease (CKD) affects 45% of adults older than 70 years of age in the US [1]. The incidence of CKD will increase significantly over the next decade due to the increasing incidence of diabetes and hypertension in the rapidly aging US population. The economic cost of CKD is staggering with Medicare spending for patients with CKD aged 65 and older exceeding $50 billion in 2013, representing 20% of all Medicare spending in this age group [2]. Contributing to the high cost of CKD is the remarkably high prevalence of cognitive impairment or overt dementia that ranges 20–50% in older patients with moderate CKD [3–8] and may reach as high as 70% in severe CKD/dialysis [9]. Cognitive impairment impacts patients negatively by contributing to functional dependence and behavioral symptoms that result in poor outcomes and decreased medication and medical care compliance. These negative consequences result in a downward spiral of functional decline and an accelerated loss of independence, which leads to premature institutionalization [10–16]. The negative impact of cognitive impairment on quality of life and emotional wellbeing is significant, and it even affects employment rates negatively [17–20]. Moreover, cognitive function for incident dialysis patients has been found to be correlated with frailty and measures of depression [21]. Additionally, it more than doubles mortality risk and increases days spent in the hospital [15, 22], contributing to the tremendous individual, societal, and economical burden of CKD. We will review the vascular milieu as it is associated with cognitive decline in patients with kidney disease and the potential therapeutic role of exercise.

## 2. Measurement of Cognitive Impairment

Cognitive impairment is commonly referred to as a reduction in global cognition that is new and affects at least 2 areas of cognitive function that can be measured using a standard cognitive function test (e.g., Mini Mental State Exam (MMSE) or the Montreal Cognitive Assessment (MOCA)) [23, 24]. Impairment can be evident in various cognitive domains: executive function (judgement and planning), language, attention, memory, and visual-spatial learning. Mild cognitive impairment (MCI) is commonly defined as a deficit in global cognition that is not consistent with aging and has not progressed to overt dementia. Although there is a lack of a consensus for a standard definition of MCI, it is known to be present with a performance of 1.5–1.99 standard deviations below the standard norm on a given cognitive test. MCI is mostly manifested in short-term memory loss but can also be manifested as impaired language and executive functions [25, 26]. Importantly, progression from MCI to overt dementia is approximately 15% per year in older patients [26]. Dementia on the other hand is used as the umbrella term for moderate/severe progressive cognitive impairment often defined as scoring 2 standard deviations below population norms in at least 2 cognitive domains [27]. Importantly, overt dementia leads to a loss of independent daily function whereas MCI does not appear to significantly affect independent daily function.

## 3. Cognitive Impairment and Dementia in CKD

It is well established that patients with kidney disease commonly have some degree of cognitive impairment and that kidney dysfunction is associated with a more rapid decline in mental function than in age matched comparisons [28, 29]. As many as 20–50% of patients with moderate CKD have established cognitive impairment or overt dementia [3–8, 30]. It should be noted that the actual population prevalence and incidence of cognitive impairment are likely underreported because published studies are primarily clinic-based and not true population studies. The United States Renal Data System Annual Data Report found a lower prevalence of cognitive impairment in CKD patients (7.6–16.8%) [22]. However, the true population prevalence is likely substantially higher. This is evidenced by Kurella et al. [6] who reported a 23–28% prevalence of cognitive impairment in stages 3-4 CKD patients (*n* = 80) seen in clinical practice and Murray et al. [9] who reported a prevalence of MCI or dementia in 87% of older dialysis patients. Most published studies have reported a prevalence of cognitive impairment of 20–50% in CKD patients and up to 70% in older patients on dialysis [3–9]. Unfortunately, less than 5% of all renal disease patients with cognitive impairment have been screened or received a medical diagnosis [9, 31]. This suggests that cognitive impairment in this group of patients is severely underdetected and not adequately addressed.

The degree of renal dysfunction appears to be correlated with the degree of cognitive impairment. Cognitive impartment has been shown in multiple studies to be associated with deteriorating renal function well before requiring dialysis, although this association is particularly strong in patients requiring dialysis [31]. Increased serum cystatin C and albuminuria are also associated with accelerated cognitive decline [32–34]. Studies have shown a 15-25% increased risk of cognitive impairment for every 10 ml/min per 1.73 m^2^ reduction in the estimated glomerular filtration rate (eGFR). Further, there is an increased odds ratio of 2.43 (95% CI 1.38 to 4.29) for cognitive impairment in patients with an eGFR of <45 ml/min/per 1.73 m^2^ even after adjustment for confounders [7, 35]. Thus, patients with CKD appear to have at least a twofold increased risk of cognitive impairment than those without CKD [7, 8, 30]. This risk increases to fourfold with further reductions in eGFR to <30 ml/min per 1.73 m^2^ independent of potential confounders [8, 35]. These findings translate to patients with CKD having an increased and accelerated risk of cognitive aging equivalent to 3.6–7 years compared to the general population [32, 36]. However, current physical examination and medical history for patients with CKD or end-stage renal disease (ESRD) do not include cognitive function measures.

In terms of the clinical implications of cognitive impairment in CKD, improving support and access to psychology and social professionals, support groups, and patient education are likely to improve outcomes, although this has yet to be determined. Support of patients with CKD should also include counseling with pharmacists and providers regarding the risk of polypharmacy and potential interactions with patient-initiated supplements. The prevalence of cognitive impairment and dementia in the growing CKD population is likely to cause strain on the healthcare system, individuals, and family members. It is imperative that clinicians recognize the risk of cognitive impairment in the CKD population and include screening for cognitive impairment and initiate prompt treatment and coping strategies.

## 4. Brain Structure in Renal Disease

Magnetic resonance imaging (MRI) techniques have been used to assess brain structure and function in patients with CKD. Older MRI techniques have shown general cerebral atrophy of the hippocampus, cortical atrophy, and prominent lesions of the frontal lobes [37–39]. More recent MRI studies have been able to show deterioration of functional structures including reduced deep white matter volume, white matter hyperintensities representing small vessel disease, white matter lesions, and overt white matter disease [40–44]. Moreover, white matter lesions (degeneration of cells in the white matter) are frequent (up to 70% in dialysis patients) in CKD patients, even before requiring dialysis, suggesting that structural alterations begin early in the CKD disease process [40–44]. White matter lesions likely reflect vascular damage and cerebral ischemic areas. Advanced MRI techniques including diffusion tensor imaging (DTI) have shown subtle alterations in brain structural connectivity of the white matter via mean diffusivity (MD) and fractional anisotropy (FA). The white matter is important for coordinating interactions between different regions of the brain and is essential for normal functioning of the brain [45–49]. Impaired white matter integrity appears to be a primary contributor to cognitive decline in CKD and is strongly affected by the internal vascular milieu [45, 46]. Several studies have reported a correlation between MD and FA values and neuropsychiatric testing for patients with CKD, on hemodialysis, and after transplant [50–52]. The use of advanced MRI measures such as DTI may provide a method to diagnose early risk of cognitive decline before symptom presentation [53]. Moreover, several newer MRI techniques are emerging such as multicomponent relaxometry techniques that may provide a tool to understand the etiology and the impact of risk factor contribution to cognitive decline in patients with renal disease [47, 50–55]. Notably, structural and functional brain changes appear to occur in conjunction with reduced cerebral blood flow, likely related to systemic and cerebral endothelial dysfunction and arterial calcification [45, 56, 57]. Interestingly, Zhang et al. (2016) attempted to evaluate potential changes in white matter integrity in a small nonrandomized single arm study by assessing patients' brain functional connectivity before and after kidney transplantation [55]. They reported that structural connectivity values were abnormal before transplantation but returned close to normal values one-month after transplantation. Radić et al. (2011) observed improvement in cognitive function following transplantation, which was maintained at 2-year follow-up [58]. The reasons for these findings are not clear and need to be confirmed in appropriately powered randomized, controlled trials. However, these studies are encouraging and suggest that the adverse brain structural changes may be susceptible to reversal although it is clear that much additional research is needed before any conclusions can be made.

## 5. Etiology of Cognitive Decline in Kidney Disease

The most common type of dementia in the general population is neurodegenerative dementia (as seen in Alzheimer's disease) often manifested as atrophy of the hippocampus. Patients with renal disease are more likely to have large and small blood vessel disease, which causes white matter disease and reduced white matter integrity related cognitive impairment that often is superimposed on neurodegenerative disease. This vascular disease results in a high rate and susceptibility of cerebrovascular disease including subclinical microvascular cerebral disease and overt stroke [59–62]. Cerebral microbleeds occur in up to 60% of all patients with CKD and appear to be more frequent in patients with black ethnicity [63, 64]. Moreover, CKD patients have a fivefold increased risk of developing clinical and subclinical cerebrovascular disease, and the annual incidence of stroke is approximately 10%, compared to 2.5% in an age and sex matched population without CKD [22, 65]. This rate is even higher in the dialysis population and may reach as high as a tenfold increased incidence of stroke compared to the general population [66, 67]. There is therefore a strong likelihood that patients with CKD are at an increased risk for cognitive impairment due to vascular disease-related causes, manifested as cerebral microinfarcts and white matter disease, and not overt Alzheimer's disease per se [68]. The cerebral vascular disease appears to act in conjunction with a neurodegenerative disease process mediated in part by uremic toxins, creatinine levels, and even cystatin C levels [62]. It should be noted that the pathophysiology and etiology of cognitive decline in CKD are complex and multifaceted, and far from completely understood.

## 6. Risk Factors Associated with Cognitive Decline in CKD

Risk factors for cognitive decline in patients with CKD are listed as follows:

*Demographic Factors*

  African American
  Hispanic
  Female sex
  Older age
  Low education

*Clinical Factors*

  Hypertension
  Diabetes
  Dyslipidemias
  Polypharmacy
  Sleep quality
  Depression

*Vascular Milieu*

  Oxidative stress
  Inflammation
  Hyperhomocysteinemia
  Uremia
  Albuminuria

*Dialysis Procedure Specific Risk Factors for Cognitive Impairment in End-Stage Renal Disease*

  Volume and electrolyte fluctuation
  Cerebral edema
  Cerebral hypoperfusion
  Hypotension during dialysis
  Excessive cytokine release
  Microembolism
  Delirium

The traditional risk factors for cerebrovascular disease include African American and Hispanic ethnicity, dyslipidemia, hypertension, diabetes mellitus, female sex, education status, and older age [7, 31, 35, 69–75]. Vascular risk factors will be discussed in detail below. Various clinical factors that are unique to the CKD population contribute to cognitive impairment. These include a high rate of undiagnosed depression and polypharmacy-related side effects or interactions [19, 20]. Patients with renal disease often have significant fatigue and daytime sleepiness related to poor sleep quality, which could contribute to further cognitive decline [76]. It should be noted that patients undergoing hemodialysis have many additional risk factors predisposing them to cognitive impairment, including the dialysis procedure itself. The dialysis procedure predisposes patients to potential risk factors for cognitive impairment and cerebrovascular disease including volume and electrolyte fluctuations, cerebral edema and hypoperfusion, and excessive cytokine release [60]. Interestingly, the frequency of hypotensive episodes during dialysis has been associated with cerebral atrophy and lacunae frequency, while microembolisms may contribute to the burden of both large and small vessel cerebrovascular disease although much research is needed in this area [77, 78]. Moreover, secondary and recurrent delirium (often related to hypoperfusion) and encephalopathy (i.e., untreated renal failure related neurotoxicity) appear to be associated with the development of cognitive impairment [79]. Finally, we recognize that anemia and derangements in serum vitamin D levels also contribute to the CKD milieu and potentially cognitive decline. In cross-sectional studies, anemia has been found to be associated with cognition in ESRD; however, in a longitudinal study, anemia was not an independent predictor of cognitive decline in elderly patients with CKD [80]. In terms of vitamin D, a review by Cheng et al. (2016) notes that reduced levels of 25(OH)-vitamin D may be contributing to cognitive impairment in CKD [81]. Clinical trials are needed to investigate the effect of vitamin D supplementation on cognitive outcomes.

## 7. The Cerebrovascular-Renal Axis (Figure 1)

The accelerated cognitive decline in older CKD patients appears to be due, in part, to the CKD disease process itself, which creates a toxic vascular and metabolic milieu that consists of chronic inflammation, oxidative stress, uremia, and systemic vascular endothelial dysfunction [82–88]. This toxic internal vascular and metabolic milieu is postulated to cause vascular dysfunction related impairment of the white matter that is superimposed on neurodegenerative damage caused by homocysteine, uremic toxins, creatinine, and cystatin C [62, 89]. Homocysteine appears to be an especially strong risk factor for stroke in CKD patients via a direct neurotoxic effect, initiation of systemic inflammation, and endothelial dysfunction [90–94]. The increase in homocysteine is probably due to reduced renal clearance. Unfortunately, interventions with folate to reduce homocysteine levels have thus far been conflicting and disappointing [95–97]. Patients with CKD have increased levels of oxidative stress, caused by uremia, production of reactive oxygen species via physiological pathways (e.g., impaired/damaged/malfunctioning mitochondria), and an inability to produce adequate antioxidative enzymes [98, 99]. These changes all contribute to a vascular milieu that consists of systemic inflammation, high levels of oxidative stress, and endothelial dysfunction that is unique to the CKD patient and creates a vascular pathway to cognitive decline.

## 8. Vascular Mechanisms Related to Cognitive Decline in CKD

Patients with CKD are at an increased risk for vascular disease-related cognitive impairment rather than Alzheimer's disease per se as described above. Vascular calcification in advanced stages of CKD, possibly including intracranial calcification, could be influencing cognition. Interestingly, there appears to be a significant influence of chronic hypertension on the progression of cognitive decline. This may be related to the high volume of blood flow and pressure that the brain and the kidney are exposed to.

The association between systemic arterial stiffness and cognitive performance has been established in cross-sectional studies [100]. More recently, Pase et al. (2016) studied the Framingham Offspring cohort and found that aortic stiffness predicts incident mild cognitive impairment and incident dementia in nondiabetic patients over 10 years [101]. Apart from aortic (central arterial) stiffness, stiffness in arteries in close proximity to the brain may need to be considered. One study reports that, in swines, carotid artery stiffness seems to be associated with impaired memory [102]. Additionally, although intracranial artery stiffness is even more challenging to measure, it may also be associated with cognitive decline [103].

Hypertension is associated with changes in brain tissue and cerebral vasculature. For example, mean arterial pressure was associated with white matter hyperintensity volume in the Framingham Offspring cohort, even in the absence of associations between changes in brain tissue and tonometry measures (such as arterial stiffness or central pulse pressure) [104]. Importantly, increased duration of hypertension is an important contributor to cognitive outcomes. Midlife hypertension has a significant impact on long-term cognitive impairment, as reviewed by Iadecola et al. (2016) [105]. Although some studies have shown a relationship between elevated blood pressure and cognitive impairment in the absence of a stroke, whether intensive hypertension control results in prevention or reversal of cognitive outcomes is unclear [106]. Upcoming results from the SPRINT-MIND trial (Systolic Blood Pressure Intervention Trial, Memory and Cognition in Decreased Hypertension) may address some of these unanswered questions.

The Strain Vessel Hypothesis states that “strain vessels” found in vital organs play a protective role [107]. Strain vessels help maintain a pressure gradient between the larger arteries and capillaries. High-pressure flow from large arteries, in addition to low resistance to flow in small vessels in vital organs, causes subsequent damage to vessels exposed to high pulsatility [108]. In the brain, small perforating arteries are exposed to high pressure; cerebral hemorrhage and infarction occur frequently in these small arteries [107, 109]. As decreasing kidney function is associated with arterial stiffness [110] and high blood pressure, patients with CKD are likely to have their blood vessels exposed to high pulsatility flow. Therefore, CKD patients are at high risk of developing injury to cerebral vasculature. The latter, in turn, would impact cognitive function.

It is challenging for drugs to influence the aorta and large arteries; and thus interventions may target other conduit arteries to reduce wave reflection. Although drugs such as angiotensin-converting enzyme inhibitors and calcium channel blockers seem to be beneficial in hypertensive elderly individuals, blood pressure levels that are optimal for cognitive function are yet to be identified [106]. Apart from medications, regular exercise may be employed to target this phenomenon.

## 9. Exercise as a Potential Therapeutic Approach

Higher levels of physical activity and cardiorespiratory fitness levels are associated with increased levels of cognitive function in healthy individuals. Exercise appears to prevent cerebral atrophy or even increase hippocampal volume in the general population [111, 112]. It is conjectured that these observations are related to an increase in brain-derived neurotrophic factor and an exercise-induced increase in angiogenesis, neurogenesis, and synaptogenesis. It is conceivable that physical activity and fitness levels are related to cognitive function in patients with kidney disease, but there have been a minimal number of studies in this area and the results are inconsistent. Patients on hemodialysis with the highest self-reported activity levels had the highest cognitive scores in one study [113]. Conversely, one smaller study found no association between maximal oxygen consumption and scores on the MMSE in patients on hemodialysis [114]. Several exercise intervention studies have shown promise in improving cognition in healthy elderly participants with and without MCI [111, 115–126], whereas others have reported no improvement [127–130]. Regular exercise and higher fitness levels in non-CKD patients with and without cognitive impairment have been associated with improved cognitive function, white matter integrity, and hippocampal volume suggesting a possible neuroprotective effect of exercise [111, 113, 115–125]. This is conjectured to be due to an improvement in vascular function-related increases in cerebral blood flow [116]. It is therefore plausible that exercise training may improve the vascular milieu and thereby contribute to improved cognitive function in patients with CKD. However, the impact of exercise on cognitive function in the CKD population is currently unknown. Only one study has reported on cognitive function following exercise training in the dialysis population. Martins et al., 2011, reported an improvement in cognitive function measured via the MMSE following exercise training [131]. Unfortunately, this study was not randomized and the MMSE is not a sensitive measure for change in global cognition, which limits any conclusions. Studies investigating the impact of exercise on cognition in CKD patients are needed. Exercise training appears to improve the vascular milieu in patients with CKD by reducing systemic inflammation and oxidative stress, arterial stiffness, and improving vascular function [132]. However, not all studies have shown improvements in these vascular risk factors, likely due to differences in sample characteristics, exercise program, and outcome measures. Moreover, exercise training may reduce traditional risk factors for cerebrovascular disease such as blood pressure and lipid profile although it should be noted that randomized controlled trials are scarce in patients with CKD and most data are based on secondary analyses from smaller trials. Exercise training is also known to improve glucose control in diabetic patients and may reduce homocysteine levels. Importantly, the pleiotropic effect of exercise provides additional benefits that are important to patients with CKD including improved quality of life, improved physical function, and reduced risk of frailty. Moreover, higher levels of physical activity have been associated with reduced risk of initiation of renal replacement therapy and higher survival rate in patients with CKD stages 3–5 [133]. Finally, emerging studies suggest that there is an independent association between prolonged sedentary time and kidney function decline, whereas higher levels of physical activity are associated with reduced levels of creatinine and lower risk of kidney impairment [134, 135]. Thus, it is plausible that exercise may affect renal function itself and thereby provide a protective effect. Despite lack of data on the impact of exercise on cognitive function, it is prudent for clinicians to recommend that patients with CKD consider initiating an exercise program and increase their daily physical activity levels to gain the mental and physical health benefits of exercise.

## 10. Summary and Conclusions

Cognitive impairment is common in patients with CKD and negatively affects health-related quality of life and other health-related outcomes. It is imperative that clinicians recognize the value of early screening for cognitive impairment and initiate preventive and treatment measures. Importantly, the decline in cognitive function appears to be multifaceted with a major involvement of vascular dysfunction in a unique CKD metabolic milieu that predisposes patients to an accelerated cognitive decline. Multidisciplinary healthcare teams are needed to provide psychosocial support and patient education on essential topics such as control of blood pressure, risks of polypharmacy, and other individualized self-care practices. Current research investigating exercise-induced improvement in cognition in non-CKD population is promising, but there are conflicting reports in the literature. As exercise training may be a plausible adjunctive therapeutic approach to improve cognitive outcomes and quality of life in patients with CKD, further research should focus on exercise as a promising approach that may retard the progression of cognitive impairment in CKD.

## Figures and Tables

**Figure 1:**
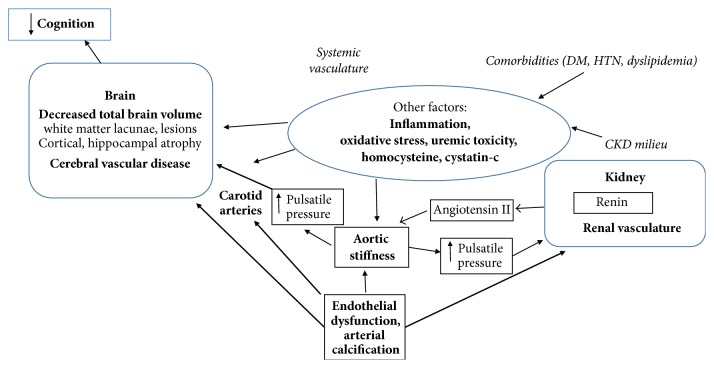
Systemic and cerebral vasculature and cognition in CKD.
